# Survival of endodontically treated teeth in public dental service in Northern Finland: a practise-based register study

**DOI:** 10.2340/aos.v83.40491

**Published:** 2024-04-25

**Authors:** Anne Laajala, Matti Nuutinen, Atso Luttinen, Hannu Vähänikkilä, Tarja Tanner, Marja-Liisa Laitala, Saujanya Karki

**Affiliations:** a Cariology, Endodontology and Paediatric Dentistry, Research Unit of Population Health, University of Oulu, City of Oulu, Finland; b Medical Research Center and Oulu University Hospital, City of Oulu, Finland; c Northern Finland Birth Cohorts, Arctic Biobank, Infrastructure for Population Studies, Faculty of Medicine, University of Oulu, Oulu, Finland; d The Wellbeing Services County of North Ostrobothnia, Oulu, Finland

**Keywords:** Endodontic, root canal therapy, survival rate, tooth extraction

## Abstract

**Objective:**

The aim of this study was to explore the factors associated with the survival of root canal treated teeth in a practise-based study setting in a 5-year period.

**Material and methods:**

This retrospective study used data from the electronic patient registration system of the public dental services of the City of Oulu, Finland. The inclusion criteria for this study were patients aged ≥ 20 years who had root canal treatment (RCT) that was initiated in 2014. One RCT per patient was included in the study. A total of 713 patients met the inclusion criteria. The outcome variable for this study was the extraction of the RCT tooth during the 5-year period. Explanatory variables included age, diagnosis, tooth type (incisive, canine, premolar, molar), RCT technique (manual, motorized), time from RCT initiation to final restoration and type of final restoration (composite, glass ionomer, fixed dental prosthesis). To evaluate the association between the outcome variable and explanatory variables, Cox regression analyses were performed.

**Results:**

The overall survival rate was 91%. The middle-aged (40–59-years-old) and the oldest (60 and older) patients had a two-fold risk of extraction compared to younger (20–40-years-old) patients. Similarly, a short length of time from RCT initiation to final restoration (0–14 days) resulted in a nearly three times higher risk of extraction compared to a longer period (≥ 90 days).

**Conclusions:**

The 5-year survival rate of RCTs seems high. Extractions were more common among patients over 40 years of age and if the RCT was completed shortly after its initiation.

## Introduction

Root canal treatment (RCT) is a procedure in which inflamed or infected dental pulp is removed, and root canals are cleaned, filled, and sealed to prevent the reinfection of the tooth and to retain the natural tooth [1]. RCTs in general perform well; their survival rate has been reported to vary between 75% and 93% depending on the follow-up time [2, 3]. The survival rates of dental implants, which seem to be an alternative to RCT in some cases, are comparable to the survival rates of RCTs [4, 5].

Endodontic treatment can be examined through the survival and success of the treated teeth. The survival of an endodontically treated tooth can be assessed as a retained natural tooth without any symptoms [6]. Success, on the other hand, usually refers to complete apical healing, evaluated both clinically and radiographically [7–9]. The difference between survival and success of RCT is essential, since a symptomless radiolucency in a functional tooth is not, by itself, reason enough to extract a RCT tooth [10]. The periapical tissues heal through complex host-derived systems. A complete healing can be considered if formation of new bone structure together with periodontal structures can be seen, while compromised healing can result in the formation of apical fibrous tissues such as granuloma, cyst or scar tissue [11]. Reasons for the compromised healing or failure of RCT include underfilling or overfilling the root canal, improper apical and coronal seal, missed root canals, perforation, ledges and broken instruments [12, 13]. Furthermore, systemic conditions of the patient (such as the presence/absence of chronic diseases, hormonal changes) may also have an affect [14, 15]. Earlier studies have shown that the patient’s socioeconomic conditions (age, gender, educational background), tooth type, instrumentation type and timing of a final restoration may also affect the survival of RCT teeth [16–21]. Alongside the healing of the RCT tooth, the retention of such a tooth in the dentition is important.

There are two dental treatment sectors in Finland: public and private. According to the Health 2011 Survey, on average 25% of adults over the age of 30 in Finland used the public dental sector (PDS) and 35% used the private dental sector [22]. This study used data from the city of Oulu, which is located in northern Finland and has a population of 200,000. Since 2019, there have been approximately 660 visits by adults in the PDS per 1,000 inhabitants in the city Of Oulu [23]. No data is available of the corresponding figures of those attending the private sector. Generally, in Finland almost two-thirds of the dentate adult population have at least one RCT tooth [24]. Of all the treatment provided in the PDS in Finland, approximately 5% was endodontic treatment in 2013 [25]. A recent study from southern Finland reported a success rate of 67% for RCTs performed by general dental practitioners [26]. Likewise, another study evaluated pre- and post-operative radiographs and reported an overall radiological success rate of 84% [27]. To our knowledge, no studies examining the survival rate of RCTs in a practise-based study setting among the Finnish population exists yet. This study was designed to examine the survival rate of RCTs conducted in the PDS. The null hypothesis was that none of the included explanatory factors are associated with the survival of root canal treated teeth.

## Materials and methods

This retrospective practise-based study was conducted in the city of Oulu, Finland. The data were extracted from the electronic patient registration system (Effica/Lifecare®) of the PDS of the city of Oulu. Data were collected in June 2020. The data included those patients who were registered according to the Finnish Institute for Health and Welfare codes indicated for root canal procedures [28]. Dental practitioners in Finland are obliged to record national treatment codes in the electronic patient system. Treatment codes indicating emergency-type as well as non-emergency RCT were used for the data collection. The inclusion criteria for this study were (1) RCT was started in 2014 or late 2013 (i.e. emergency visit in late 2013) and (2) age ≥ 20 years in 2014. No radiographs were utilized since the intention was only to examine tooth survival. All dentist professionals performing endodontic treatments were included.

**Figure 1:**
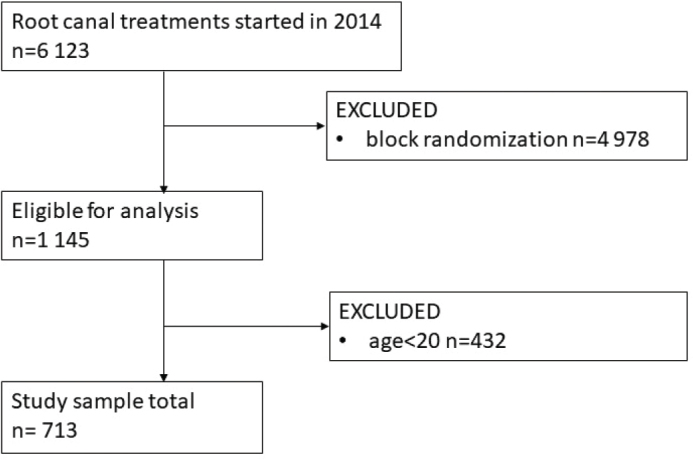
Flow chart of the study population formation.

To achieve sufficient power for this study, a goal of at least *n* = 322 teeth was set for the sample size (confidence level α = 0.05 and power 1-β = 0.80; 67% hypothesized survival [26]). First, a chief of PDS in the city of Oulu screened the patient register system according to the endodontic treatment codes mentioned above. A total of 6,123 RCTs were carried out during the study period. Then, every fifth page of the resulted listing was printed and delivered to researchers containing a total of 1,145 patient records. Patients under 20 years of age were excluded from the study first. After that each record was checked by hand and altogether 713 of them met the inclusion criteria (Figure 1). If there were multiple RCTs started for one person, the first one was included in this study. All recordings of RCT teeth included in the study were followed up till June 2020, resulting in a maximum of 5.5 years of follow-up time.

The outcome variable was extraction of the tooth (failure) at the 5-year period. All other situations of the RCT tooth were considered survival. Clinical and radiological healing of RCT were not evaluated. The explanatory variables for this study were age (20–39 years, 40–59 years, & ≥ 60 years), gender (male & female), RCT type (primary & secondary), number of dentists involved in treatment (1 clinician, 2 clinicians & ≥ 3 clinicians), education level of dentists involved (undergraduate dental student, general practitioner, specialized dentist), number of visits taken for RCT completion (1–2 visits, 3–4 visits & ≥ 5 visits), days from the first RCT visit until the root canal filling (0–14 days, 15–90 days & > 90 days), days from the first RCT visit until the final restoration of the tooth (0–30 days, 31–90 days & > 90 days), diagnosis to initiation of RCT (ICD 10 codes & written in patient register), instrumentation type (hand instrumentation & rotary instrumentation) and tooth types (incisors/canines, premolars & molars). If the tooth was re-treated by RCT within the follow-up time, it was registered. The extraction date was recorded if a RCT tooth was extracted. The reasons for extraction were not available.

### Ethical considerations

Scientific research using a patient register is subject to Finnish legislation (Act 552/2019), and permission to use a patient register for academic purposes is granted by the register administrator. Additional ethical approval is not required. As this study utilized an electronic patient registration system from the city of Oulu, permission to use the database was obtained from the Health Services Committee of the City of Oulu, Finland (Permission number: OUKA/6980/07.01.04.02/2020). The data were anonymized during data gathering and patient records were accessed only once per RCT.

### Statistics

Data collected for this study were transferred to the SPSS software (version 26, SPSS, Inc., Chicago, IL, USA) for statistical analyses. During analyses, missing values were not taken into account.

Descriptive statistics were computed as mean, standard deviation (SD), and proportions. To assess the difference in proportion between groups, a chi-square test and Fischer’s exact test were used. To evaluate the association between the outcome variable and co-variates, Cox regression analyses were performed. Both unadjusted and adjusted hazard ratios (HR) and 95% confidence intervals (95% CI) were computed. The model was adjusted only for those variables that were significant during univariate analyses. The Kaplan–Meier survival curve was drawn to compare the survival of an RCT in 5 years by age groups and RCT duration. The significance threshold was set at 0.05.

## Results

The number of RCT teeth as well as participants in this study was *n* = 713. The mean age of the study participants was 41.7 (SD 14.2) years, and the study population was slightly dominated by females (54.1%). The mean RCT duration was 142 (SD 179) days. The overall survival rate was 91.3% and a total of 62 teeth were extracted during the five-year period. The mean survival rate for primary RCT was 91.6% and for the secondary RCT 87.5%. A statistically significant difference was found among age groups (*p* = 0.011) and RCT duration (*p* = 0.013), the older age groups having more RCT teeth extracted compared to younger ones, and teeth treated in a shorter time range were extracted more often than those whose treatment took a longer time (Table 1). Only 2% (*n* = 16) of the teeth in this study were re-treated during follow-up and only two of those were extracted. No statistically significant difference was found between re-treated RCT teeth and those RCT teeth not re-treated during follow-up (*p* = 0.642).

**Table 1:** Characteristics of the study participants stratified by survival and extraction of root canal treated tooth.

Factor	Survived *n* (%) teeth	Extracted *n* (%) teeth	*p*
**Age group (years)**			
20–39	329 (94.5)	19 (5.5)	0.011^a^
40–59	251 (88.4)	33 (11.6)	
≥ 60	71 (87.7)	10 (12.3)	
**Gender**			
Male	295 (90.2)	32 (9.8)	0.353^a^
Female	356 (92.2)	30 (7.8)	
**Number of visits**			
1–2	50 (92.6)	4 (7.4)	0.342^a^
3–4	435 (92.2)	37 (7.8)	
≥ 5	166 (88.8)	21 (11.2)	
**RCT type**			
Primary	602 (91.6)	55 (8.4)	0.318^b^
Secondary	49 (87.5)	7 (12.5)	
**Number of dentists involved**			
1	327 (91.6)	30 (8.4)	0.365^a^
2	225 (89.6)	26 (10.4)	
≥ 3	99 (94.3)	6 (5.7)	
**Dentist’s education level**			
General dental practitioner	525 (92.3)	44 (7.7)	0.185^a^
Specialist	61 (88.4)	8 (11.6)	
Undergraduate dental student	65 (86.7)	10 (13.3)	
**Root canal treatment duration (days from initiation to root canal filling)**			
0–14	39 (79.6)	10 (20.4)	0.013,^a^
15–90	268 (91.8)	24 (8.2)	
≥ 91	340 (92.4)	28 (7.6)	
**Final restoration (days from root canal filling)**			
0–30	343 (90.5)	36 (9.5)	0.731^a^
31–90	166 (91.7)	15 (8.6)	
≥ 91	138 (92.6)	11 (7.4)	
**Tooth type**			
Incisor/canine	109 (89.3)	13 (10.7)	0.167^a^
Premolar	213 (94.2)	13 (5.8)	
Molar	329 (90.1)	36 (9.9)	
**Diagnosis** ^+^			
Pulpitis	203 (93.5)	14 (6.5)	0.309^a^
Necrosis or apical periodontitis	234(90.7)	24 (9.3)	
**Instrumentation type**			
Hand instrumentation only	85 (88.5)	11 (11.5)	0.324^a^
Motorized instrumentation	481 (91.8)	43 (8.2)	

a Chi-square test;

b Fischer’s exact test;

+ Diagnosis was missing in *n* = 238 cases.

In most of the cases (*n* = 671, 94.1%), calcium hydroxide paste (AH temp® [Dentsply Sirona, Konstanz, Germany] or Ultracal XS® [Ultradent, South Jordan, USA]) was used as the intra-canal medication, while the rest used a mixture of corticosteroid and antibiotics (Maxitrol®, Novartis Finland, Helsinki, Finland). All the teeth were temporarily sealed between appointments with temporary filling (materials recorded were Cavit® (3M Espe AG, Seefeld, Germany), Fuji II LC^®^ (GC Europe, Leuven, Belgium), Fuji IX^®^ (GC Europe, Leuven, Belgium), IRM® (Dentsply Sirona, Konstanz, Germany), Coltosol® (Coltene/Whaledent AG, Altstätten, Switzerland), Ionostar® (VOCO GmbH, Cuxhaven, Germany), Equia Forte® (GC Europe, Leuven, Belgium)). For root canal filling, gutta-percha was used with sealer, recorded sealers were AH+® (Dentsply Sirona, Konstanz, Germany), EndoRez® (Ultradent, South Jordan, USA), SealApex® (ORMCO B.V./SybronEndo, BR Amersfoort, Netherland) and MTAFillapex® (Angelus dental product industry, Londrina, Brasil). Most often AH+® was used (74.9%, *n* = 643). The teeth were finally restored with direct composite restorations or fixed dental prostheses. Fixed dental prosthesis was prepared for *n* = 20 (2.8%) RCT teeth.

In the unadjusted model, both middle-aged patient group (40–59 years old) and the oldest age group (60 years and over) had double the risk of tooth extraction compared to the youngest age group (20–39 years). The shortest RCT duration (0–14 days) had an almost three times higher risk of extraction compared to a longer RCT duration (> 90 days). When the model was adjusted, the risk remained unchanged for the middle-aged patient group (40–59 years old) and for the shorter RCT duration (0–14 days) (Table 2).

**Table 2:** Factors associated with the survival of root canal treatment computed by cox regression analyses.

Factor	Model 1 HR (95% CI)	Model 2 aHR (95% CI)
**Age (years)**		
20–39	Ref.	Ref.
40–59	2.19 (1.25–3.86)	2.20 (1.25–3.86)
≥ 60	2.40 (1.12–5.17)	2.00 (0.91–4.39)
**RCT duration (days from RCT initiation to roof canal filling)**		
≥ 91	Ref.	Ref.
0–14	2.88 (1.40–5.92)	2.64 (1.25–5.78)
15–90	1.10 (0.64–1.89)	1.06 (0.61–1.83)
**Gender**		
Female	Ref.	-
Male	1.28 (0.78–2.10)	-
**Number of visits**		
1–2	Ref.	-
3–4	1.05 (0.37–2.93)	-
≥ 5	1.51 (0.52–4.40)	-
**RCT types**		
Primary	Ref.	-
Secondary	1.51 (0.69–3.33)	-
**Re-RCT during follow-up**		
No	Ref.	-
Yes	1.49 (0.36–6.10)	-
**Number of dentists involved**		
≥ 3	Ref.	-
1	1.54 (0.64–3.69)	-
2	1.91 (0.79–4.63)	-
**Types of clinician involved**		
General dental practitioner	Ref.	-
Specialist	1.53 (0.72–3.24)	-
Dental student	1.79 (0.90–3.56)	-
**Final restoration (days from root canal filling to permanent restoration)**		
≥ 91	Ref.	-
0–30	1.32 (0.67–2.59)	-
31–90	1.15 (0.53–2.50)	-
**Tooth types**		
Incisor/Canine	Ref.	-
Premolar	0.51 (0.24–1.10)	-
Molar	0.91 (0.48–1.71)	-
**Diagnosis**		
Pulpitis	Ref.	-
Necrosis	1.51 (0.78–2.91)	-
**Instrumentation types**		
Motorized instrumentation	Ref.	-
Hand instrumentation	1.42 (0.73–2.76)	-

HR (95% CI): Hazard ratios (95% confidence intervals); aHR (95% CI): adjusted hazard ratios (95% confidence interval); Model 1 analysis was done as crude model; Model 2 analysis was done as adjusted model for age, and root canal filling duration.

**Figure 2:**
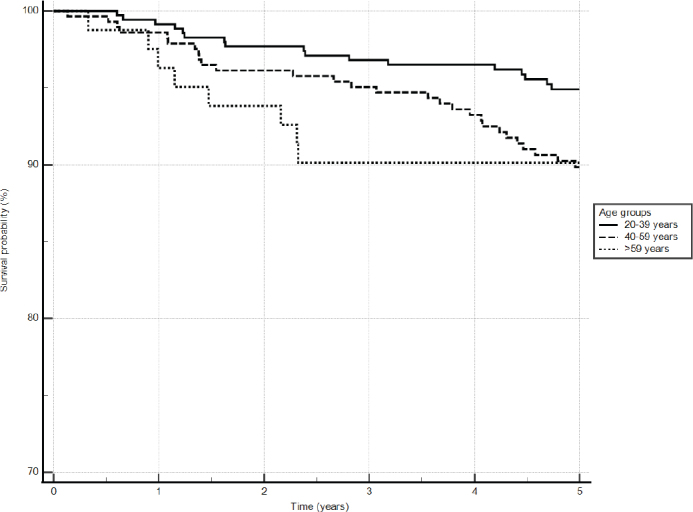
Survival curve by age groups using Kaplan–Meier survival analysis. The adjusted *p* = 0.011.

**Figure 3:**
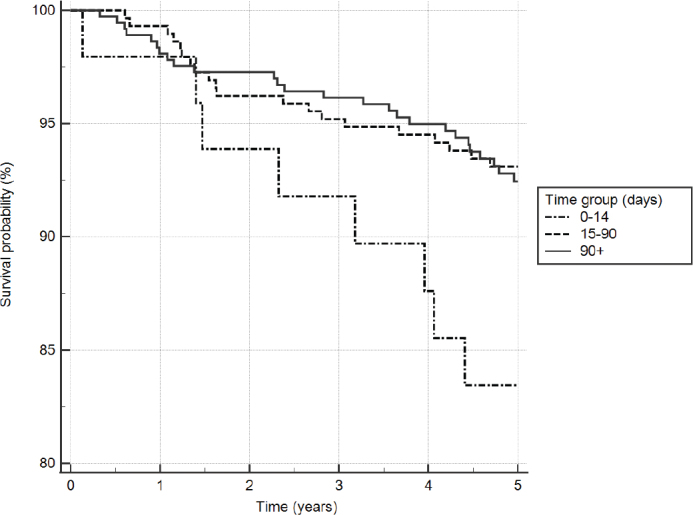
Survival curve by root canal treatment duration using Kaplan–Meier survival analysis. The adjusted *p* = 0.013.

The Kaplan–Meier survival analysis showed that the youngest age group had the highest survival rate (94.5%), while the survival rate of the two older age groups was lower (88.4% for 40–59-years old, 87.7% for 60-years old and older) at the 5-year follow up (Figure 2). Likewise, the longest RCT duration (> 90 days) had the highest survival rate (93.5%) (Figure 3).

## Discussion

This study examined the survival of RCT teeth after 5 years of RCT initiation. The data represent a practise-based setting in the Northern Finland. A patient’s young age and a longer RCT duration were associated with a high survival rate of RCT. The null hypothesis was partly rejected.

This retrospective study is the first of its kind in Finland to explore factors associated with the survival of RCT teeth. One strength of this study is the randomization done for the selection of patients, decreasing the possibility of selection bias. The study data were collected from PDS records, which enables the results to be applicable to at least the PDS in the city of Oulu, Finland. Neither the dentists nor the patients were aware at the time of the RCT that their registers would be included in this study 5 years afterward. The PDS in Finland is organized and legislated the same way in all the regions of the nation, even though some individual and regional differences may exist.

One of the study limitations is that it was not possible in this study to explore patient records of any other dental sectors of Oulu or other Finnish districts than the aforementioned register of the city of Oulu. Another limitation was that missing variables were not taken into account because the database contains only information about the procedures that the dentist had completed during the visit. Another shortcoming is the lack of radiographs, which could have enabled the assessment of radiological changes in apical bone as well as the visual quality of the root canal filling. In addition, no follow-up examinations clinically or by questionnaire were obtained. The retained RCT teeth in the data may include teeth with symptoms or other conditions, but the patients have not sought treatment in the PDS of Oulu.

In this study, the 5-year survival rates for both primary and secondary RCT were relatively high, 92% on average. A similar study was conducted in Sweden where the 10-year survival rate of RCT was 82%, which is in concordance with our study, since the survival of RCT teeth tend to decrease over time [29]. Survival rates of 88% to as high as 98% for RCT have been previously reported [19, 26]. In Mareschi’s study [30], RCT was performed solely by one specialist and for this reason the results cannot be compared as such with this practise-based study. The absence of radiographs may not change the good survival rate here, because even teeth with a minor symptomless periapical lesion can be retained for decades [31]. There are non-infectious reasons for apical radiolucency after RCT, such as apical healing by scar tissue formation and cysts [32], which would not have been healed with RCT alone. Therefore, assessing the healing of an RCT tooth by only interpreting apical radiolucency may lead to unnecessary tooth extractions. In this register-based study it was not possible to examine the symptoms of RCT teeth. Studies on the health consequences of not treating a tooth with periapical radiolucency are needed.

In this study, the youngest age group (20–39 years) had the highest survival rate compared to other age groups. A similar finding has been reported earlier in other populations [17, 19, 33]. It can be argued that because tissue repair slows with age [34], it might cause difficulties in assessing the healing of periapical tissues and thus lead to unnecessarily early extraction of RCT teeth compared to younger age groups. In addition, there might have been both oral conditions (such as periodontal disease, caries and missing teeth) and general health conditions, especially among older age groups, that have favored extraction over prolonged follow-up of RCT. However, there are studies that conclude that increased patient age alone does not influence the prognosis of RCT [35]. To rule out these findings, further investigation is necessary, as the reason for the extraction of RCT tooth and possible comorbid conditions were not studied here.

The Finnish Current Care guidelines of RCT state that RCT could be completed at one appointment or within 1–2 weeks depending on the diagnosis and reason for RCT [36]. Calcium hydroxide eliminates microorganisms in the intracanal space most when it is kept there for at least 7 days [37]. However, some tooth groups may benefit from a multiple visit RCT [38]. In this study, the average time to complete an RCT was over 4 months. A treatment time longer than 2 weeks had a higher survival rate than those whose RCT was completed within 2 weeks after the first visit. There is no explanation for why the treatment time in this study was so long, but a similar trend was found in Sweden, where the duration of treatment was also nearly 4 months [29]. In both studies, ours and Kebke’s [29], the long RCT duration was not associated with poor tooth survival. In addition, the length of time from root canal filling to placement of a final restoration was not associated with RCT survival here. The number of fixed dental prostheses as a final restoration was very low, which can be a reason why those did not elevate the RCT survival rate. A recent practise-based study on RCT survival did, however, find both the timing of the final restoration and fixed dental prostheses to affect survival rate [3]. A shortage of dentists in the PDS in Finland might be one reason for the prolonged RCT duration, as even the general waiting times from first contact to a visit to a dentist in the PDS of Finland range from 3 to 6 months [39]. The reason for the lower survival rate of those RCTs, which were completed within 0–14 days is unclear. The reasons for this are probably a combination of multiple factors, including time spent to perform an RCT, duration of intracanal medication and chemical and mechanical disinfection repetitions despite the initial status of the pulp (i.e. vital or not).

From the patient’s point of view, the results of this study are encouraging. Almost all RCT teeth become a part of the dentition after 5 years. For a dentist in the PDS, the results provide much needed information, namely that RCT teeth perform well even when the RCT cannot be completed within the recommended time span.

## Declarations of interests

The authors state that they have no conflict of interests.

## Authors’ contributions

Conceptualization: ALa, MLL; data acquisition: Ala ALu, MN; methodology: ALa, MLL, HV; data analysis and interpretation: ALa, ALu, MN, VH, SK; writing-reviewing: ALa, ALu, MN, HV, TT, MLL, SK; editing: ALa, TT, MLL, SK; supervision: ALa, SK.

## Data availability

The data that support the findings of this study are available from the corresponding author upon reasonable request.
